# The associations between whole grain and refined grain intakes and serum C-reactive protein

**DOI:** 10.1038/s41430-021-00996-1

**Published:** 2021-08-17

**Authors:** Riikka E. Taskinen, Sari Hantunen, Tomi-Pekka Tuomainen, Jyrki K. Virtanen

**Affiliations:** University of Eastern Finland, Institute of Public Health and Clinical Nutrition, Kuopio, Finland

**Keywords:** Risk factors, Epidemiology

## Abstract

**Background/objectives:**

Epidemiological studies suggest that whole grain intake has inverse associations with low-grade inflammation, but findings regarding refined grains are inconclusive. Our objective was to investigate whether consumption of whole or refined grains is associated with serum high sensitivity CRP (hs-CRP).

**Subjects/methods:**

The study included 756 generally healthy men and women aged 53–73 years from the Kuopio Ischaemic Heart Disease Risk Factory Study, examined in 1999–2001. Dietary intakes were assessed using 4-day food records. ANCOVA and linear regression were used for analyses.

**Results:**

The mean intake of whole and refined grains was 136 g/day (SD 80) and 84 g/day (SD 46), respectively. Higher whole grain intake was associated with lower hs-CRP concentration and higher refined grain intake with higher concentration after adjustment for lifestyle and dietary factors. Each 50 g/d higher whole grain intake was associated with 0.12 mg/L (95% Cl 0.02–0.21 mg/L) lower hs-CRP concentration and each 50 g/d higher refined grain intake with 0.23 mg/L (95% Cl 0.08–0.38) higher concentration. Adjustment for fibre from grains attenuated the associations especially with whole grains. There were no statistically significant interactions according to gender or BMI (P for interactions >0.065).

**Conclusions:**

The results of this study suggest that higher intake of whole grains is associated with lower concentrations of hs-CRP and higher intake of refined grains is associated with higher concentrations. However, especially the association with whole grain intake was attenuated after adjusting for fibre intake from grains, suggesting that cereal fibre may partly explain the association.

## Introduction

Low-grade inflammation is a state characterized by elevated concentrations of inflammatory markers such as C-reactive protein (CRP) [1]. Chronic, low-grade inflammation has been acknowledged as a risk factor for a range of chronic diseases, such as type 2 diabetes [2], coronary heart disease [3] and cancer [4]. Low-grade inflammation is also linked to other diseases, such as dementia and depression [5, 6] and to all-cause mortality [7]. It is also thought to mediate the impact of excessive adipose tissue on pathophysiology of different chronic diseases [8, 9].

A growing body of evidence indicates that greater consumption of whole grain products is associated with lower risk of cardiovascular disease, type 2 diabetes and certain cancers [10], metabolic syndrome [11] and mortality [7]. Previous observational studies have presented an inverse relationship between whole grain consumption and low-grade inflammation [12–15], but available data from randomized clinical trials is conflicting [16–19]. Studies have also presented inconsistent findings about the relationship between refined grain consumption and low-grade inflammation. The evidence regarding health outcomes of refined grain consumption is also inconsistent, with most studies reporting negative or neutral effects regarding low-grade inflammation [13, 19] and disease outcomes [20, 21]. Refined grains have, however, been associated with unhealthy diet patterns that may have an unfavourable effect on risk of chronic diseases [22, 23].

To elucidate the impact of the type of grain products on low-grade inflammation, we examined cross-sectional relationships between whole grain and refined grain consumption and serum high-sensitive CRP (hs-CRP) concentration, a marker of low-grade inflammation, among generally healthy elderly men and women from the Kuopio Ischaemic Heart Disease Risk Factor Study (KIHD).

## Materials and methods

### Study population

KIHD is an on-going population-based study designed to investigate risk factors for cardiovascular diseases and other chronic diseases in middle-aged men and women from eastern Finland. The study protocol was approved by the Research Ethics Committee of the University of Kuopio. All subjects gave their written informed consent.

The baseline examinations of the KIHD were conducted in 1984–1989 (Supplementary Figure). A total of 2682 men who were 42, 48, 54 or 60 years old at baseline and living in the city of Kuopio and neighbouring rural communities were recruited in two cohorts. The first cohort consisted of 1166 men who were 54 years old, enroled in 1984–1986, and the second cohort included 1516 men who were 42, 48, 54 or 60 years old, enroled in 1986–1989. In 1998−2001 all men from the second cohort were invited to the 11-year re-examinations, and 854 men participated. At these examinations also a random sample of 920 postmenopausal women from the same area, aged 53–73 years, entered the study. Of those eligible, 85.6% of men and 78.4% of women participated. The data from these 11-year examinations were used in the current analyses.

We excluded from the analyses participants who had data missing on serum hs-CRP or on dietary intakes or who had serum hs-CRP > 10 mg/L or blood leucocyte count >11 × 10^9^/L, indicating an acute inflammation. In addition, we excluded participants with a disease with inflammatory component: rheumatoid arthritis, colitis, diabetes, claudication, ischaemic heart disease, cardiac insufficiency, stroke, cancer, or disease of gall bladder, liver or pancreas. The final number of participants in the analyses was 391 men and 365 women.

### Measurements

The subjects gave fasting blood samples between 8 and 10 AM. They were instructed to abstain from ingesting alcohol for three days and from smoking and eating for 12 h prior to giving the sample. Detailed descriptions of the determination of serum lipids and lipoproteins, blood glucose, assessment of medical history and medications, family history of diseases, smoking, alcohol consumption and blood pressure have been published [24]. Education was assessed in years by using self-administered questionnaire. Annual income was obtained from a self-administered questionnaire. Diabetes was defined as self-reported diabetes mellitus or fasting blood glucose of ≥6.7 mmol/L. Physical activity was assessed using the KIHD 12-Month Leisure-Time Physical Activity Questionnaire that covers the type, frequency, duration and intensity of the activity [25]. Body mass index (BMI) was computed as the ratio of weight in kilograms to the square of height in metres, both measured during the study visit.

### Serum hs-CRP

Serum hs-CRP concentrations were measured at the time of the examinations in 1998-2001 with an IMMULITE chemiluminescent immunoassay system (Diagnostic Products Corporation, Los Angeles, USA). The lower detection limit of the assay, defined as the concentration two standard deviations above the response at zero dose, is 0.1 mg/l and the functional sensitivity (coefficient of variation <20%) was 0.2 mg/L.

### Dietary assessment

Dietary intakes were assessed using a 4-day food record of three weekdays and one weekend day at baseline. Participants received instructions on how to complete the food records from a nutritionist using conventional household measures. To aid with portion-size estimates, participants received a book with pictures of 126 common foods and dishes. The book contained pictures of 3–5 commonly used portion sizes for each food item. The participant could also describe the portion size in relation to the examples in the book. The completed food records were cross-checked by a nutritionist together with the participants to minimize reporting bias.

Food and nutrient intakes were estimated as the mean intake over the four days from the food records using the NUTRICA® 2.5 software (Social Insurance Institution, Turku, Finland). The databank of the software is mainly based on Finnish values of nutrient composition of foods. Butter included butter as such and butter in butter-vegetable oil margarine mixes. Whole grain was defined according to the HEALTHGRAIN definition as the whole kernel of grain or cereal [26]. The whole grain variable represents the total whole grain intake, including whole grains (e.g., whole grain flour, whole grain pasta) in mixed dishes and recipes. The intake of refined grains was calculated by subtracting the amount of whole grains from the total grain intake.

### Statistical analysis

ANCOVA and linear regression were used for analyses. For ANCOVA, homogeneity of group variances was confirmed. The analyses were controlled for possible confounders, which were selected based on previously reported relations with serum CRP or on relations with outcomes or exposures in the current study. Four models were developed. Model 1 was adjusted for age (y), gender, energy intake (kcal/d) and the year of examination. Model 2 included the variables in model 1 plus BMI (kg/m^2^), pack-years of smoking, leisure-time physical activity (kcal/day), years of education and intakes of alcohol (g/week). Model 3 included variables in model 2 plus dietary variables including fat quality (ratio of saturated + trans fatty acids to polyunsaturated + monounsaturated fatty acids) and intakes of fruits, vegetables and berries (g/day), red meat (g/day), dairy (g/day), fish (g/day), butter (g/day), vegetable oil margarine (g/day), and eggs (g/day). Model 4 included variables in model 3 plus fibre from grains. Missing values in covariates were replaced with the cohort mean (*n* = 48 in annual income, *n* = 9 in pack-years of smoking, *n* = 1 in alcohol intake). Statistical significance of the potential interactions by gender and BMI (<25, 25-<30 and ≥30 kg/m^2^) was assessed by stratified analysis and likelihood ratio tests using a multiplicative interaction term. Linear trends across quartiles were assessed after assigning the median value for the grain intake quartiles and then treating that as a continuous variable in the statistical models. Normality of distributions was checked using histograms. All *P* values were 2-tailed (*α* = 0.05). SPSS 25 for Windows (Armonk, NY: IBM Corp.) was used for analyzing the data.

## Results

The mean intake of whole grains was 164 g/day (SD 92) in men and 106 g/day (SD 51) in women and the mean intake of refined grains was 92 g/day (SD 51) in men and 75 g/day (SD 39) in women. The correlation coefficient between the whole grain and refined grain intake was 0.04 (*P* = 0.448) in women and −0.10 (*P* = 0.043) in men. The mean serum hs-CRP concentration was 1.77 mg/L (SD 1.7) in men and 1.96 mg/L (SD 1.8) in women.

Table 1 shows the baseline characteristics according to whole grain and refined grain intakes. Those with higher whole grain intake were more likely to be men, not have hypertension, have lower BMI and healthier diets, including higher intakes of fibre, fish, dairy, vegetable oil margarines, fruits, vegetables and berries. They also had higher intakes of energy, butter and red meat. Those with higher intakes of refined grains were more likely to be men, have higher leisure-time physical activity, lower alcohol intake and higher intakes of energy, fish, dairy, vegetable oils and margarines, and fruits, berries and vegetables. Higher intakes of both whole and refined grains were associated with lower use of anti-inflammatory medications.

**Table 1:** Characteristics of 756 participants in the Kuopio Ischaemic Heart Disease Risk Factor (KIHD) Study in 1999–2001, according to grain intake.

	Whole grains				Refined grains			
	Intake quartile (intake range, g/d)				Intake quartile (intake range, g/d)			
	1 (<84)	2 (84–117)	3 (118–163)	4 (>163)	1 (<50)	2 (51–77)	3 (78–109)	4 (>109)
Number of subjects	189	189	189	189	189	189	189	189
Age, years	60.9 ± 6.3	61.6 ± 6.5	61.7 ± 6.1	60.8 ± 6.2	61.8 ± 6.4	61.6 ± 6.5	60.9 ± 6.2	60.9 ± 6.1
Men, %	33	33	58	83	44	47	50	66
Body mass index, kg/m^2^	27.8 ± 4.8	26.5 ± 3.7	26.5 ± 3.5	26.3 ± 3.2	27.4 ± 4.1	26.6 ± 4.0	26.4 ± 4.0	26.8 ± 3.4
Waist circumference, cm	91.1 ± 13	87.7 ± 11.9	90 ± 10.6	92.1 ± 10.1	91.2 ± 11.4	89 ± 12.1	89.3 ± 12.5	91.4 ± 10.1
Leisure-time physical activity, kcal/d	170 ± 163	197 ± 276	198 ± 194	187 ± 176	180 ± 160	161 ± 170	181 ± 164	231 ± 297
Education, years	10.4 ± 3.5	9.8 ± 3.7	9.8 ± 3.4	9.6 ± 3.3	10.0 ± 3.8	10.1 ± 3.7	9.6 ± 3.1	10.0 ± 3.3
Current smoker, %	14	12	12	13	16	11	10	13
Hypertension, %	57	60	49	41	59	49	43	55
Regular use of anti-inflammatory medication, %	10	7	7	5	8	10	6	5
Serum LDL cholesterol, mmol/L	3.8 ± 1.0	3.7 ± 0.9	3.7 ± 0.9	3.6 ± 0.8	3.8 ± 0.9	3.6 ± 0.9	3.7 ± 0.9	3.7 ± 0.9
Serum HDL cholesterol, mmol/L	1.3 ± 0.3	1.3 ± 0.3	1.3 ± 0.3	1.2 ± 0.3	1.3 ± 0.3	1.3 ± 0.3	1.3 ± 0.3	1.2 ± 0.3
Serum triglycerides, mmol/L	1.2 ± 0.7	1.2 ± 0.6	1.2 ± 0.6	1.1 ± 0.6	1.1 ± 0.5	1.1 ± 0.6	1.2 ± 0.6	1.2 ± 0.7
Alcohol intake, g/wk	63 ± 151	56 ± 93	49 ± 103	55 ± 82	61 ± 102	66 ± 153	54 ± 106	42 ± 56
*Dietary intakes*								
Energy, kcal/d	1572 ± 508	1694 ± 372	1921 ± 455	2408 ± 520	1609 ± 508	1835 ± 510	1926 ± 511	2225 ± 556
Protein, E%	17.2 ± 3.2	17.2 ± 2.7	17.0 ± 2.9	16.8 ± 2.8	17.8 ± 3.1	17.3 ± 2.9	16.6 ± 2.6	16.4 ± 2.8
Carbohydrates, E%	45.8 ± 7.3	46.6 ± 6.6	48.1 ± 6.8	47.8 ± 6.5	45.6 ± 8.0	46.3 ± 6.3	48.1 ± 6.4	48.1 ± 6.2
Fibre, g/d	17.8 ± 5.5	20.8 ± 4.8	22.1 ± 5.2	27.5 ± 8.4	23.3 ± 7.5	22.2 ± 7.1	21.9 ± 7.1	21.0 ± 6.4
Total fat, E%	34.3 ± 6.2	33.7 ± 5.6	33.0 ± 5.5	33.6 ± 5.9	33.9 ± 6.4	33.8 ± 5.8	33.1 ± 5.4	33.8 ± 5.6
Polyunsaturated fatty acids, E%	4.9 ± 1.4	4.9 ± 1.5	5.0 ± 1.5	4.8 ± 1.2	4.9 ± 1.5	4.9 ± 1.5	4.7 ± 1.3	5.0 ± 1.3
Monounsaturated fatty acids, E%	11.3 ± 2.7	10.9 ± 2.5	10.7 ± 2.3	10.6 ± 2.2	10.9 ± 2.6	10.9 ± 2.5	10.6 ± 2.3	11.0 ± 2.2
Saturated fatty acids, E%	14.5 ± 3.2	14.2 ± 3.1	13.8 ± 3.0	14.4 ± 3.7	14.4 ± 3.6	14.4 ± 3.2	14.2 ± 3.1	14.1 ± 3.3
Trans fatty acids, E%	0.9 ± 0.4	0.9 ± 0.4	0.9 ± 0.3	1.0 ± 0.4	0.9 ± 0.4	0.9 ± 0.3	0.9 ± 0.4	1.0 ± 0.4
Butter, g	8.8 ± 9.2	9.8 ± 10.8	11.5 ± 12.7	19.7 ± 21.8	10.9 ± 14	11.5 ± 15.1	13.7 ± 14.7	13.7 ± 16.4
Fish, g/d	38 ± 47	43 ± 50	45 ± 53	60 ± 78	47 ± 61	45 ± 59	43 ± 54	52 ± 61
Dairy, g/d	400 ± 239	463 ± 234	484 ± 267	569 ± 270	425 ± 248	454 ± 246	505 ± 262	531 ± 270
Vegetable oils, g/d	3.0 ± 4.0	3.0 ± 4.0	3.7 ± 5.0	3.2 ± 4.9	2.1 ± 3.0	3.3 ± 4.3	3.1 ± 4.2	4.4 ± 5.8
Vegetable oil margarines, g/d	13.8 ± 10.4	15.1 ± 13.0	16.8 ± 13.7	20.8 ± 19.6	11.7 ± 11.1	14.8 ± 13.0	16.4 ± 12.7	23.5 ± 18.7
Red meat, g/d	97 ± 83	92 ± 64	105 ± 71	134 ± 85	147 ± 81	146 ± 80	137 ± 70	152 ± 75
Fruits, berries and vegetables, g/d	298 ± 172	301 ± 146	293 ± 155	307 ± 171	290 ± 165	298 ± 155	301 ± 171	309 ± 152

Values are means ± SD or percentages.

Table 2 illustrates the serum hs-CRP concentrations in quartiles of whole grain and refined grain intakes. In model 1 adjusted for age, gender, examination year and energy intake, whole grain intake was associated with statistically significantly lower concentrations of hs-CRP (difference between the highest and the lowest quartile 0.76 mg/L, 95% CI 0.34–1.18 mg/L). Evaluated continuously, each 50 g/d higher intake was associated with 0.17 mg/L (95% CI 0.08–0.27 mg/L) lower hs-CRP concentration. Further adjustment for lifestyle (model 2) and dietary factors (model 3) attenuated the associations. Adding fibre from grains into the model attenuated the association even further (model 4) and the associations were not statistically significant anymore.

**Table 2:** Concentrations of serum C-reactive protein (mg/L) in quartiles of grain and fiber intakes.

	Exposure quartile					
	1 (*n* = 189)	2 (*n* = 189)	3 (*n* = 189)	4 (*n* = 189)	*P* for trend	CRP change per 50 g higher grain intake
Whole grains, g/d	<84	84-117	118-163	>163		
Model 1	2.30 (2.03–2.56)*	1.84 (1.59–2.10)	1.77 (1.52–2.02)	1.53 (1.25–1.82)	0.001	−0.17 (−0.08 to −0.27)
Model 2	2.12 (1.85–2.36)	1.85 (1.61–2.30)	1.83 (1.59–2.06)	1.66 (1.38–1.93)	0.059	−0.12 (−0.03 to −0.21)
Model 3	2.12 (1.85–2.37)	1.85 (1.60–2.09)	1.82 (1.58–2.05)	1.67 (1.39–1.95)	0.060	−0.12 (−0.02 to −0.21)
Model 4	2.03 (1.74–2.33)	1.82 (1.57–2.07)	1.82 (1.58–2.05)	1.77 (1.43–2.12)	0.450	−0.07 (0.11 to −0.25)
Refined grains, g/d	<50	51–77	78–109	>109		
Model 1	1.89 (1.63–2.15)	1.61 (1.36–1.86)	1.93 (1.68–2.17)	2.02 (1.76–2.28)	0.226	0.14 (−0.01 to −0.29)
Model 2	1.76 (1.51–2.01)	1.60 (1.37–1.84)	1.99 (1.75–2.23)	2.09 (1.84–2.34)	0.017	0.24 (0.10–0.38)
Model 3	1.78 (1.53–2.03)	1.61 (1.38–1.85)	1.97 (1.73–2.21)	2.08 (1.82–2.33)	0.032	0.23 (0.08–0.38)
Model 4	1.82 (1.56–2.07)	1.62 (1.38–1.86)	1.96 (1.73–2.20)	2.04 (1.78–2.30)	0.085	0.20 (0.05–0.35)
Fibre from grains, g/d	<11	11-13	14–17	>17		CRP change per 5 g higher fibre intake
Model 1	2.16 (1.90–2.41)	1.94 (1.69–2.20)	1.74 (1.49–1.99)	1.60 (1.35–1.86)	0.001	−0.17 (−0.07 to −0.27)
Model 2	2.09 (1.85–2.34)	1.87 (1.63–2.11)	1.81 (1.57–2.04)	1.67 (1.43–1.91)	0.027	−0.11 (−0.01 to −0.21)
Model 3	2.08 (1.83–2.33)	1.90 (1.66–2.14)	1.79 (1.55–2.03)	1.67 (1.42–1.91)	0.023	−0.11 (−0.01 to −0.22)
Total fibre g/d	<18	18–21	22–25	>25		
Model 1	2.35 (2.11–2.60)	1.98 (1.73–2.22)	1.43 (1.18–1.68)	1.68 (1.43–1.93)	<0.001	−0.19 (−0.10 to −0.28)
Model 2	2.25 (2.01–2.50)	1.99 (1.76–2.28)	1.47 (1.23–1.71)	1.73 (1.49–1.97)	0.001	−0.13 (−0.04 to −0.22)
Model 3	2.22 (1.96–2.48)	1.99 (1.75–2.22)	1.47 (1.23–1.71)	1.77 (1.51–2.03)	0.008	−0.12 (−0.02 to −0.22)

^*^Values are means (95% confidence interval).

Model 1 adjusted for age, gender, examination year and energy intake.

Model 2 adjusted for variables in Model 1 plus body mass index, pack-years of smoking, leisure-time physical activity, years of education and intakes of alcohol.

Model 3 adjusted for variables in Model 2 plus fruits, vegetables and berries, fat quality, red meat, dairy, fish, butter, vegetable oil margarine, and eggs.

Model 4 adjusted for variables in Model 3 plus fibre from grains.

Fibre intake from grains was associated with lower serum hs-CRP concentrations (Table 2). For example, each 5 g/d higher intake was associated with 0.11 mg/L lower (95% CI 0.01–0.21 mg/L) hs-CRP concentrations (Model 3). Similar inverse associations were observed with total fibre intake (Table 2).

Higher refined grain intake was not associated with serum hs-CRP-concentration in the model 1 but was associated with higher concentration in the multivariable-adjusted models 2 and 3 (for example, in the model 3, the difference in the highest vs. the lowest quartile was 0.30 mg/L, 95% CI −0.08–0.68 mg/L). Each 50 g/d higher intake was associated with 0.23 mg/L (95% CI 0.08–0.38 mg/L) higher hs-CRP concentration. Further adjustment for fibre from grains slightly attenuated the association (model 4).

**Fig. 1:**
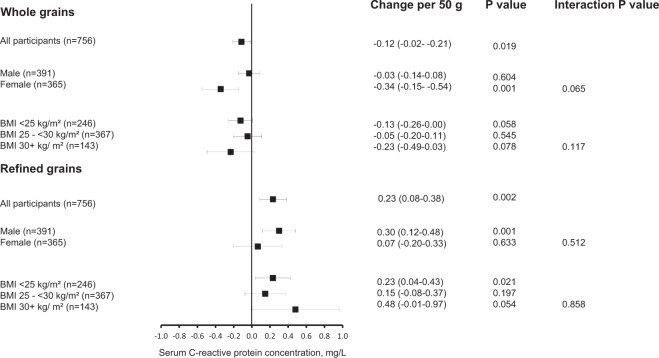
Association of whole grain and refined grain intakes with serum C-reactive protein concentration, according to gender and body mass index. The black box indicates the mean change in the serum C-reactive protein concentration for each 50 g/d higher grain intake and the error bars indicate the 95% confidence interval for the mean.

In the stratified analyses based on gender and BMI, the multivariable-adjusted (model 3) associations were in general similar in all three BMI categories (Fig. 1). In contrast, although the interactions were not statistically significant, whole grain intake had a stronger inverse association with hs-CRP among the women (P-interaction = 0.065) and refined grain intake among the men (P-interaction = 0.512).

## Discussion

In this population-based cohort among Finnish elderly men and women, higher intake of whole grains was associated with lower concentrations of hs-CRP and higher intake of refined grains with higher hs-CRP. BMI did not modify these associations, but there was indication that the inverse association with whole grains was stronger in women and that the direct association with refined grains was stronger in men.

Our results regarding whole grain intake and hs-CRP are concordant with previous observational studies. In a cross-sectional study of 902 diabetic women, consumption of whole grains and cereal fibre was associated with lower CRP and tumour necrosis factor receptor 2 (TNF-R2) levels [14]. Two other cross-sectional studies supported these results [12, 15]. In a recent cross-sectional study of 2689 adults, whole grain intake was associated with lower CRP concentrations but as in our study, the association was attenuated to nonsignificant after adjusting for cereal fibre intake [27]. Another study in a sample of 5496 men and women demonstrated an inverse relationship between whole grain consumption and CRP levels, but after adjusting for BMI and insulin levels the association was no longer significant [28]. Similarly, a study of 983 healthy men failed to demonstrate a significant association between intakes of whole grains, bran and germ and CRP, interleukin-6 (IL-6) or fibrinogen [29].

Contrary to our results, in a study of Masters et al. [13]. refined grain intake was not associated with CRP concentrations. The authors found, however, that refined grain intake was positively associated with another pro-inflammatory protein, plasminogen activator inhibitor-1 (PAI-1), which suggests that refined grains may have pro-inflammatory effects. In our study, higher intake of refined grains was associated with higher hs-CRP concentrations in models adjusted for lifestyle and dietary covariates, suggesting that the association is independent of these factors.

Data on the association between grain consumption and low-grade inflammation in intervention studies is somewhat conflicting. A recent meta-analysis of 14 randomized controlled trials did not find a significant effect of whole grain intake on serum concentrations of CRP, IL-6, TNF-alpha or PAI-1 [30], whereas in another meta-analysis of 17 randomized controlled trials, whole grain intake resulted in significantly lower concentrations of serum hs-CRP and IL-6 but not TNF-alpha, compared with refined grain intake [31].

The incoherence of results in studies investigating grain product consumption and low-grade inflammation might result from various issues. First, there is inconsistency in the amount of grain products consumed in these studies. For example, in our study, the mean whole grain intake was 136 g/day, which is notably higher than for example in the study of Jensen et al. (22 g/day). The types of grain products may also differ. Although we did not have information on the proportional contribution of different foods to grain intake in our study, in the national FinDiet Study in 2002 rye bread, porridge and mixed-flour bread accounted for about 2/3 of the grain intake [32]. These foods contain mostly whole grains. Buns, biscuits, and sweet or savory bakeries, which mostly contain refined grains, accounted for 1/5 of the grain intake. There is also variation in the dietary assessment methods, with one study using a 24-h dietary recall [15] while many others used a food frequency questionnaire [13, 14, 28, 29]. We used a food record, which has not been used in previous cross-sectional studies. Lastly, it must be noted that the CRP levels in the study populations differ. In the present study, the mean hs-CRP concentrations were <2 mg/L, whereas for example in the studies of Qi et al. and Lutsey et al., the mean CRP was higher, 5.75 mg/L and 3.26 mg/L, respectively.

Epidemiological studies have presented evidence of the inverse relationship between dietary fibre and inflammatory markers, mainly CRP [33, 34]. While majority of existing research concentrates on total dietary fibre intake and its association with inflammation, few studies have discussed the effect of different sources of dietary fibre on inflammatory markers. Our results regarding the association between cereal fibre intake and hs-CRP are consistent with some, but not all previous studies. For example, in the study of Qi et al. [14]. higher intake of cereal fibre was inversely associated with concentrations of CRP and TNF-R2. In another study, a statistically significant inverse association was found between cereal fibre and a cluster of pro-inflammatory cytokines, including IL-6 and TNF-α [35]. Barrett et al. [27] found that intake of cereal fibre was borderline inversely associated with CRP-levels. Contradictory, in a study of Gibson et al. [36] the intake of whole grain fibre and cereal non-whole grain fibre was associated with lower serum CRP concentrations in a crude model, but not in the adjusted model. In our study the addition of fibre from grains to the models attenuated especially the relationship between whole grain intake and hs-CRP. This suggests that the high fibre content in grain products may at least partly explain the inverse association of whole grain intake with low-grade inflammation.

There are various mechanisms that could explain the beneficial effect of dietary fibre intake on low-grade inflammation. One possible mechanism is the decrease of lipid oxidation, which in turn may help lower inflammation [33]. Other possible mechanisms include the anti-inflammatory effect of fibre intake’s ability to prevent weight gain, and the beneficial impact on glycemic control [37]. Anti-inflammatory mechanisms unrelated to body weight include the alteration of intestinal microbiome due to fibre intake, resulting in changes in the immune system and bacterial metabolites in the intestine, thus modulating the inflammatory response locally as well as systemically [38]. The protective effect of whole grains has been attributed to several different biologically active components besides fibre, such as phytochemicals, vitamins, minerals and unsaturated fatty acids, as well as phytoestrogens and antioxidative properties [39, 40]. It has been proposed that dietary fibre acts as a transporter of bioactive components through the gastrointestinal tract, thus making dietary fibre a link between the bioactive components and health [41].

There is an inverse relationship between whole grains and BMI and waist circumference according to observational studies [42]. Adipose tissue releases many inflammatory proteins and cytokines, and the concentrations of these are known to be higher among obese individuals than non-obese [9, 43]. CRP levels are also known to correlate positively with BMI and waist circumference [8]. Thus, there is a possibility that BMI could mediate the association between whole- and refined grains and low-grade inflammation. In one observational study the association between whole grains and inflammatory markers attenuated to non-significant after adjusting for BMI [28], suggesting that body weight might explain some of the association, while in other studies BMI did not alter the results [12, 15, 29]. However, in our analyses BMI did not seem to modify the associations.

Strengths of this study include the population-based recruitment, inclusion of both men and women and a large number of potential confounders. This enabled us to, for example, exclude participants that had a disease with an inflammatory component. Dietary intakes were assessed with food recording, which is considered a gold standard in assessing dietary intakes in population studies. As grain products are commonly consumed daily or almost daily, four days of recording can be assumed to be enough to accurately assess grain intake. This study also has limitations. Due to the cross-sectional and observational design, it is impossible to determine the causality of the investigated factors. Hs-CRP was the only inflammatory marker measured in this study, and at the moment there is no consensus regarding the best biomarker to represent low-grade inflammation [1]. The study population largely consists of elderly Caucasians located in a geographically exclusive area, limiting the generalizability of the results. Since whole grains are a significant part of healthy lifestyle and diet [22], it is possible that this study only partly accounted for potential confounding factors, resulting in overestimating the

impact of whole grains on low-grade inflammation. This applies to refined grains as well, as these have been associated with unhealthier dietary patterns [22, 23]. However, in our study neither whole grain intake nor refined grain intake was systematically associated with better or worse lifestyle or dietary factors that could explain our findings.

In conclusion, higher intake of whole grains was associated with a lower serum hs-CRP concentrations and higher refined grain intake with higher concentrations in an elderly population from eastern Finland. These findings reinforce the current public health and nutrition recommendations that whole grain products should be preferred [44]. Further investigation is needed to determine potential pathways between grain products and low-grade inflammation. Finally, well-designed intervention studies on the effect of grains and their components on low-grade inflammation are warranted.

## Supplementary information

The timeline of the Kuopio Ischaemic Heart Disease Risk Factor Study

## Data Availability

Code availability can be inquired by contacting the corresponding author.
