# Supplementary material for: The associations between whole grain and refined grain intakes and serum C-reactive protein
Source: Eur J Clin Nutr. 2021 Aug 17;76(4):544–50. doi: 10.1038/s41430-021-00996-1 (PMC8993682; doi:10.1038/s41430-021-00996-1)
Supplement: Supplementary file 1 — The timeline of the Kuopio Ischaemic Heart Disease Risk Factor Study [file 41430_2021_996_MOESM1_ESM.pdf]

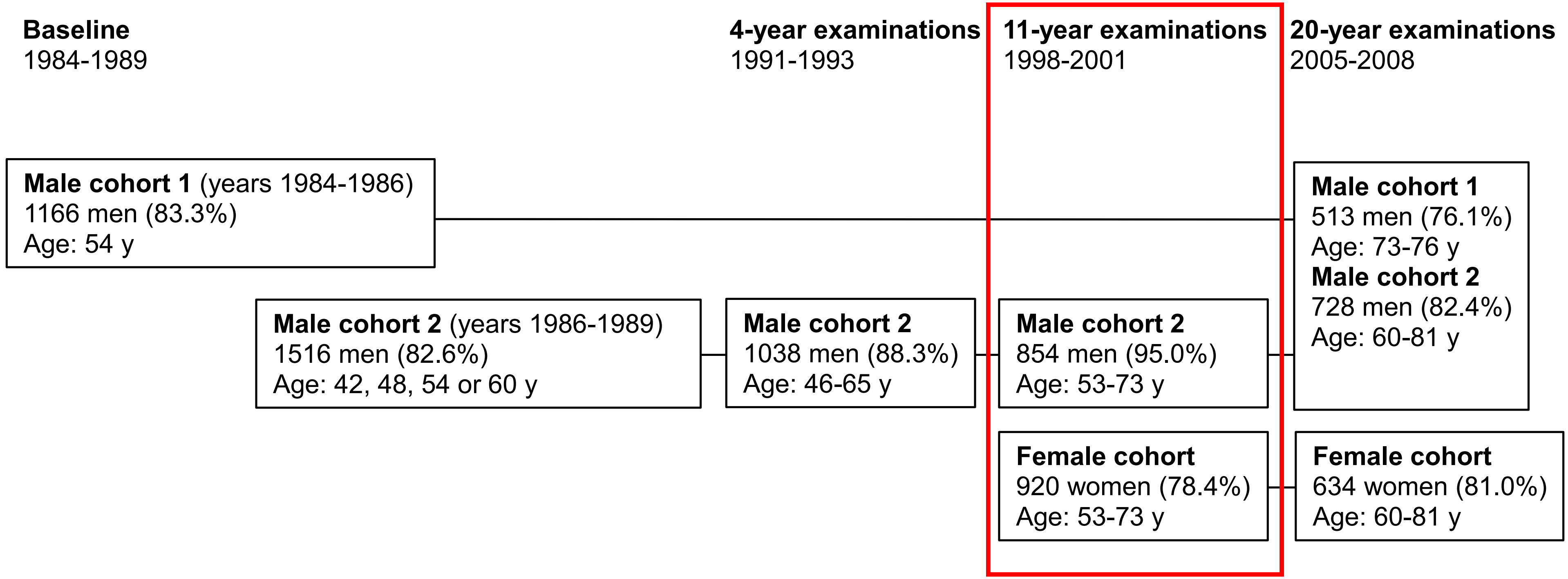

Supplemental Figure. The timeline of the Kuopio Ischaemic Heart Disease Risk Factor Study (KIHD). Percentages in brackets indicate the proportion of the eligible participants that participated in the study visits. The red box indicates the examinations that were used for the cross-sectional analyses in the current study.
